# 

**Published:** 1857-07

**Authors:** 


### LIST OF COLLABORATORS.

John L. Atlee, M.D., President Pennsylvania State Medical Society.
Walter F. Atlee, M.D., Philadelphia.
John Bell, M.D., Philadelphia, formerly Professor of Medicine, Medical College of Ohio.
T. S. Bell, M.D., Louisville, Ky.
S. M. Bemiss, M.D., Louisville, Ky.
L. P. Blackburn, M.D., Natchez, Miss.
G. C. Blackman, M.D., Professor of Surgery, Medical College of Ohio.
W. S. Bowen, M.D., New York.
J. L. Bobbs, M.D., formerly Professor of Anatomy, Indianapolis.
W. M. Boling, M.D., Montgomery, Ala., formerly Professor of Midwifery, Transylvania University, Lexington, Ky.
N. Bozeman, M.D., Montgomery, Ala.
J. T. Bradford, M.D., Augusta, Ky.
John H. Brinton, M.D., Lecturer on Operative Surgery, Philadelphia.
W. S. Chipley, M.D., Professor of Medicine, Transylvania University.
A. Clapp, M.D., New Albany, Ia.
D. B. Cliffe, M.D., Franklin, Tenn.
J. Da Costa, M.D., Lecturer on the Practice of Medicine, at the Philad. Med. Association.
M. Dupierris, M.D., Havana, Cuba.
W. F. Edgar, M.D., United States Army.
S. C. Farrar, M.D., Jackson, Miss.
C. S. Fenner, M.D., Memphis, Tenn.
Austin Flint, M.D., Professor of Clinical Medicine and Pathology, University of Buffalo.
Charles Fricke, M.D., Baltimore.
W. Y. Gadbury, M.D., Benton, Miss.
O. C. Gibbs, M.D., Chautauque County, New York.
Dr. J. P. Hall, Glasgow, Ky.
John Hardin, M.D., Professor of Obstetrics, Kentucky School of Medicine, Louisville.
Edward Hartshorne, M.D., One of the Surgeons to Wills Hospital for Diseases of the Eye, Philadelphia.

E. B. HASKINS, M.D., Clarksville, Tenn., late President of the Tennessee State Medical Society.

C. A. HENTZ, M.D., Marianna, Florida.

ADDINELL HEWSON, M.D., One of the Surgeons to Wills Hospital for Diseases of the Eye, Philadelphia.

SAMUEL L. HOLLINGSWORTH, M.D., late Editor of the Medical Examiner, Philadelphia.

WILLIAM A. HAMMOND, M.D., United States Army.

WILSON JEWELL, M.D., Philadelphia, President of the Quarantine and Sanitary Convention.

G. A. KUNKLER, M.D., Madison, Ia.

WM. V. KEATING, M.D., Lecturer on Obstetrics and the Diseases of Women, in the Philadelphia Medical Association.

BEN. LOGAN, M.D., Shreveport, La.

A. LOPEZ, M.D., Mobile, Ala., formerly President of the Alabama State Medical Society.

GEORGE R. MOREHOUSE, M.D., Philadelphia.

H. WEIR MITCHELL, M.D., Lecturer on Physiology at the Philad. Med. Association.

B. I. RAPHAEL, M.D., New York.

J. C. REEVE, M.D., Dayton, Ohio.

L. C. RIVES, M.D., formerly Professor of Midwifery, Medical College of Ohio.

J. LAWRENCE SMITH, M.D., Professor of Chemistry, University of Louisville.

W. C. SNEED, M.D., Frankfort, Ky., President, Kentucky State Medical Society.

C. H. SPILMAN, M.D., Harrodsburg, Ky., late President of Kentucky State Medical Society.

GEO. SUTTON, M.D., Aurora, Ia.

W. L. SUTTON, M.D., Georgetown, Ky., formerly President Kentucky State Medical Society.

HORATIO R. STORER, M.D., One of the Physicians to the Boston Lying-in Hospital, Boston, Mass.

S. THOMPSON, M.D., Albion, Ill.

E. WILLIAMS, M.D., Cincinnati, Ohio.

### Bi-Monthly Bibliographical Record.

### AMERICAN.

Catalogue Raisonné of the Medical Library of the Pennsylvania Hospital. By Emil Fischer, M.D., Printed by order of the Board of Managers, 8vo. pp. 750. Philadelphia, 1857. T. K. & P. G. Collins. (Received from the Hospital.)

Indigenous Races of the Earth; or New Chapters of Ethnological Inquiry; including Monographs on Special Departments of Philology, Iconography, &c. &c., contributed by Alfred Maury, Francis Pulszky, and J. Aitken Meigs, M.D. Presenting fresh Investigations, Documents, and Materials. By J. C. Nott, M.D., and George R. Gliddon. 8vo. pp. 640. J. B. Lippincott & Co., Philadelphia, 1857. (Received from the Publishers.)

A Manual of Examinations upon Anatomy, Physiology, Surgery, Practice of Medicine, Chemistry, Materia Medica, Pharmacy, and Therapeutics, especially designed for Students of Medicine. By J. L. Ludlow, A.M., M.D., Fellow of the College of Physicians, &c. &c. A New Edition, thoroughly revised and much enlarged. 12mo. pp. 816. Blanchard & Lea, Philadelphia, 1857. (Received from the Publishers.)

Catalogue of Human Crania in the Collection of the Academy of Natural Sciences of Philadelphia. Based upon the Third Edition of Dr. Morton's "Catalogue of Skulls," &c. By J. Aitken Meigs, M.D., Librarian of the Academy, &c. &c. Pamphlet, pp. 112. J. B. Lippincott & Co., Philadelphia, 1857. (Received from the Author.)

The Transactions of the Academy of Science of St. Louis. With plates illustrating papers. Vol. I. Pamphlet, pp. 86. St. Louis, 1857.

Investigations, Chemical and Physiological, relative to certain American Vertebrata. By Joseph Jones, M.D., Professor of Chemistry in the Savannah Medical College. 4to. pp. 137. G. P. Putman & Co., New York, 1857. [From the Smithsonian Contributions to Knowledge.] (Received from the Author.)

Researches into the Structure and Functions of the Kidney. By C. E. Isaacs, M.D., Demonstrator of Anatomy in the University of the City of New York. Pamphlet. pp. 85. (Republished from the Transactions of the New York Academy of Medicine by S. S. & W. Wood, 1857.)

Annual Rep ort of the Physician-in-Chief of the Marine Hospital at Quarantine, New York. Presented to the Legislature of the State of New York. Pamphlet, pp. 63, Albany, 1857.

### FOREIGN.

On the Diseases of Women; including those of Pregnancy and Childbed. By Fleetwood Churchill, M.D., F.C.D., M.R.I.A., &c. &c. A New American Edition, revised by the Author. With Notes and Additions by D. Francis Condie, M.D. 8vo. pp. 768. Republished by Blanchard & Lea, Philadelphia, 1857. (Received from the Publishers.)

A Manual of the Detection of Poisons by Medico-Chemical Analysis. By Dr. Fr. Jul. Otto, Professor of Chemistry in Caroline College, Brunswick. Translated from the German, with Notes and Additions, by Wm. Elderhorst, M.D., Professor of Chemistry, Rensselaer Polytechnic Institute, Troy, N. Y. 12mo., pp. 178. H. Bailliere, New York, 1857.

The Functions and Disorders of the Reproductive Organs, in Youth, in Adult Age, and in Advanced Life, considered in their Physiological, Social, and Psychological Relations. By W. Acton. 8vo. London, 1857.

The Constitution of the Animal Creation as expressed in Structural Appendages. By G. C. Holland. 8vo. London, 1857.

The Hygienic Treatment of Pulmonary Consumption. By B. W. Richardson, M.D. 8vo. London, 1857.

Leçons sur la Physiologie et l'Anatomie Comparée de l'Homme et des Animaux, faites a la Faculté des Sciences a Paris. Tome Ier. 8vo. 1857.

Compressed Air as a Therapeutic Agent in the Cure of Consumption, Asthma, Chronic Bronchitis, and other Diseases. By A. Simpson, M.D., &c. &c. 8vo. Edinburgh, 1857.

Practical Observations on Mental and Nervous Disorders. By A. B. Maddock. 8vo. 2d edition. London, 1857.

